# Autoantibody-associated psychiatric syndromes in children: link to adult psychiatry

**DOI:** 10.1007/s00702-021-02354-8

**Published:** 2021-05-31

**Authors:** Niels Hansen, Daniel Luedecke, Berend Malchow, Michael Lipp, Jonathan Vogelgsang, Charles Timäus, Tristan Zindler, Stefan Gingele, Simone Kühn, Jürgen Gallinat, Klaus Wiedemann, Johannes Denk, Nicole Moschny, Jens Fiehler, Thomas Skripuletz, Christian Riedel, Mike P. Wattjes, Inga Zerr, Hermann Esselmann, Luise Poustka, Anne Karow, Hans Hartmann, Helge Frieling, Stefan Bleich, Jens Wiltfang, Alexandra Neyazi, + CAP (Cerebrospinal Fluid Analysis in Psychiatry)-Consortium

**Affiliations:** 1 Department of Psychiatry and Psychotherapy, University Medical Center Goettingen, Von-Siebold-Str. 5, 37075 Göttingen, Germany; 2 Department of Psychiatry, Social Psychiatry and Psychotherapy, Hannover Medical School, Carl-Neuberg Str. 1, 30625 Hannover, Germany; 3 Department of Psychiatry and Psychotherapy, University Hospital Hamburg-Eppendorf, Martinistr. 52, 20251 Hamburg, Germany; 4 Department of Neurology, Hannover Medical School, Carl-Neuberg Str. 1, 30625 Hannover, Germany; 5 Department of Neurology, University Medical Center Göttingen, Robert-Koch Str. 40, 37075 Göttingen, Germany; 6 Department of Neuroradiology, University Medical Center Göttingen, Robert-Koch Str. 40, 37075 Göttingen, Germany; 7 Department of Neuroradiology, University Hospital Hamburg-Eppendorf, Martinistr. 52, 20251 Hamburg, Germany; 8 Department of Neuroradiology, Hannover Medical School, Carl-Neuberg Str. 1, 30625 Hannover, Germany; 9 Department of Childhood and Adolescence Psychiatry, University Medical Center Göttingen, Robert-Koch Str. 40, 37075 Göttingen, Germany; 10 Clinic for Pediatric Kidney, Liver and Metabolic Diseases, Hannover Medical School, Carl-Neuberg Str. 1, 30625 Hannover, Germany; 11 German Center for Neurodegenerative Diseases (DZNE), Von-Siebold-Str. 3a, 37075 Göttingen, Germany; 12 Neurosciences and Signaling Group, Department of Medical Sciences, Institute of Biomedicine (iBiMED), University of Aveiro, Aveiro, Portugal

## Abstract

Studies show that psychiatric symptoms in adults and children are sometimes associated with serum neural autoantibodies. The significance of serum neural autoantibodies associated with psychiatric symptoms in children remains often unclear, but might be relevant for the extent and occurrence of psychiatric disease manifestation in later life, as well as therapy and outcome. For this narrative review, we sought articles listed in PubMed and published between 1988 and 2020 addressing the maternal–fetal transfer of neural autoantibodies and psychiatric disorders associated with serum neural autoantibodies. We identified six major subgroups of psychiatric disorders in children that are associated with serum neural autoantibodies: patients with attentional deficit hyperactivity disorder, autism spectrum disorder, obsessive compulsive disorder, Gilles de la Tourette syndrome, psychosis and catatonia. Furthermore, we summarized study findings from maternal–fetal transfer of Contactin*-*associated protein*-*like 2, *N*-methyl-d-aspartate receptor and fetal brain autoantibodies associated with behavioral effects in animals and humans. We hypothesize that the maternal transfer of serum neuronal autoantibodies during or after birth could result (1) in the ignition of an autoimmune-mediated inflammation having neurodevelopmental consequences for their children (autoimmune-priming-attack hypothesis) and (2) has a potential impact on the later manifestation of psychiatric disorders. Through this narrative review, we propose a diagnostic pathway for the clinical diagnosis of a potentially autoimmune origin of psychiatric symptoms in children while considering recent guidelines.

## Introduction

**Fig. 1:**
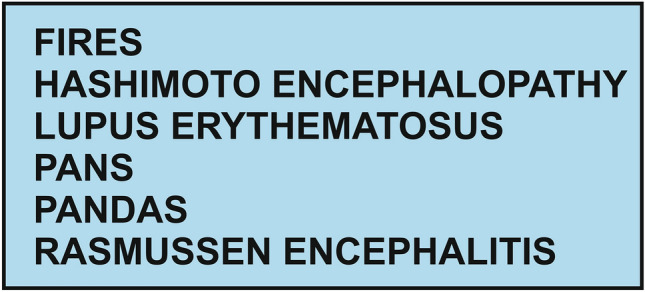
Important differential diagnoses limited to autoimmune pediatric disorders. The following differential diagnoses of pediatric autoimmune disorders have to be considered in prior to assume a pediatric autoantibody-associated psychiatric syndrome. *FIRES* febrile infection-related epilepsy syndrome, *PANS* pediatric acute-onset neuropsychiatric syndrome, *PANDAS* pediatric autoimmune neuropsychiatric disorders associated with streptococcal infections

Autoimmune-mediated encephalitis in children manifests with an acute or subacute neuropsychiatric syndrome concomitant with paraclinical findings and/or underlying neuronal autoantibodies lasting less than three months (Cellucci et al. 2020). Pediatric autoimmune encephalitis is characterized in particular by features such as prodromal fever, multifocal seizures, and a relapse-remitting course in addition to the typical features of autoimmune encephalitis also observed in adults ranging from seizures to psychiatric abnormalities and memory disturbances (Cellucci et al. 2020). The psychiatric symptom-spectrum often comprises the symptoms such as a stereotypical behavior, hyperactivity, hypersexuality, insomnia, mood dysfunction, psychosis or mild behavioral changes (Cellucci et al. 2020; Hacohen et al. 2013; Titulaer et al. 2013; Armangue et al. 2015; Florance et al. 2009). Neural autoantibodies detected in patients with pediatric autoimmune encephalitis can be divided into antibodies against membrane surface antigens such as anti-*N*-methyl-d-aspartate receptor (NMDAR), anti-myelin oligodendrocytic glycoprotein (MOG) antibodies, and antibodies against intracellular antigens such as anti-glutamic acid decarboxylase 65 (GAD65) antibodies. Much more seldom are the following cell-surface antibodies identified in children with autoimmune encephalitis: antibodies against the anti-dopamine 2 (DR2) receptor, gamma aminobutyric acid A/B receptor (GABAA/B)-receptor, glycin-receptor and metabotropic glutamate receptor 5 (mGluR5) (Cellucci et al. 2020). The latest evidence suggests that only those antibodies that target cell-surface antigens are pathogenic, but not those against intracellular antigens. Pediatric autoimmune encephalitis should be distinguished from the clinical diagnosis of PANS (pediatric acute-onset neuropsychiatric syndrome) (for differential diagnosis, Fig. 1), which is characterized by an abrupt onset of obsessive compulsive disorder (OCD) or heavily restricted food intake and more than two additional symptoms in terms of anxiety, aggression, behavioral, sensory or motor abnormalities or sleep dysfunction (for review, see Murphy et al. 2014). The PANS criteria were introduced to describe syndromes with a suspected trigger (e.g., infectious or environmental), and modified by the PANDAS (Pediatric Autoimmune Neuropsychiatric Disorders Associated with Streptococcal Infections) criteria, which entail a tic disorder also, and prior streptococcal infections with antibodies against streptococcal proteins, human brain enolase or neural tissue (Nicollini et al. 2015; Shimasaki et al. 2020) or calcium/calmodulin-dependent protein (CaM) kinase II activity (Kirvan et al. 2003, 2006; Chain et al. 2020). Co-occurring antinuclear antibodies (ANA) and elevated anti-thyroid antibodies have been detected in some patients with diagnosed PANS (Gromark et al. 2019). However, it is currently debatable whether PANS and PANDAS are independent disease entities that can be separated from tics or OCD (Gilbert et al. 2019). Furthermore, other rare childhood diseases should be considered as differential diagnoses, such as Rasmussen encephalitis, a neuroinflammation limited to one brain hemisphere causing severe cognitive dysfunction and drug-resistant epilepsy (Varadkar et al. 2014) (Fig. 1). In addition, FIRES (febrile infection-related epilepsy syndrome) is another childhood epilepsy syndrome involving febrile infection preceding seizures (Hon et al. 2018). Hashimoto encephalopathy is another autoimmune condition in children that is essential to consider, as it encompasses an encephalopathy-causing cognitive dysfunction and neuropsychiatric symptoms due to thyroid autoantibodies (anti-thyreoglobulin and anti-thyroid peroxidase) (Mattozzi et al. 2020). Furthermore, it is advisable to screen for autoantibodies associated with systemic lupus erythematosus entailing neuropsychiatric features, such as anti-ds DNA (double-stranded desoxyribonucleic acid antibodies) and anti-antiphospholipid antibodies in children, as psychiatric syndromes such as depression and anxiety often accompany childhood-onset lupus erythematosus (Quilter et al. 2019).

**Fig. 2:**
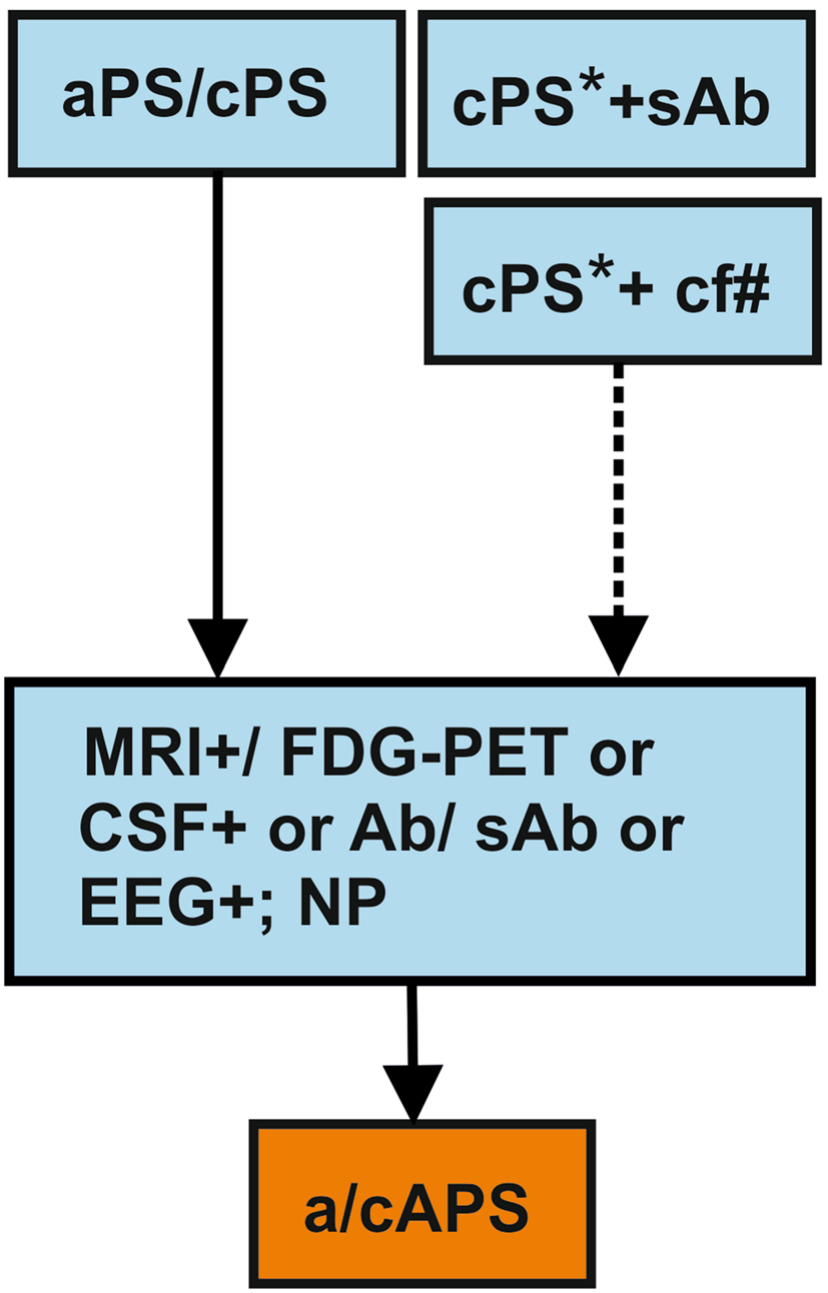
Diagnostic pathway for the diagnostics of pediatric neural autoantibody-associated psychiatric symptoms. Figure illustrates a simplified diagnostic pathway. Consider in particular subacute (aPS) or subchronic (cPS) psychiatric syndrome with the following suspected diagnosis subgroups [* = schizophrenia spectrum disorders, obsessive–compulsive disorder, autism spectrum disorders, attention deficit hyperactivity disorder, Gilles de la Tourette’s syndrome/tic disorder, catatonia] and one symptom from the following symptom cluster [# = psychosis, obsessive–compulsive, autistic or impulsive behavior, catatonia and sleep dysfunction]. Differential diagnoses encompassing other disease entities involving the immune system must be thoroughly considered (Fig. 1). If prior diagnostics have already been done in cPS (cPS*), serum neural autoantibodies (sAb) or ≥ 2 clinical features (Table 2) justify additional diagnostics (EEG, MRI/FDG-PET or CSF). *Ab* neural autoantibodies, *a/c APS* subacute or subchronic autoimmune psychiatric syndrome, *a/c PS* subacute or subchronic psychiatric syndrome, *CSF* cerebrospinal fluid, *EEG* electroencephalography, *NP* neuropsychological testing, *FDG-PET* fluorodesoxyglucose positron emissions tomography, *MRI* magnetic resonance imaging, *sAb* serum neural autoantibodies. *Means cPS with prior diagnostics (EEG, MRI, CSF). cPS* + *sAb/cf* cPS with prior diagnostics and presence of serum neural autoantibodies or clinical features. *MRI* +  MRI suggestive of encephalitis, *CSF* + pleocytosis in CSF, *EEG* +  focal or generalized epileptic potentials or slowing, *cf#* =≥ 2 clinical features

Besides these pediatric syndromes, psychiatric symptomatology may be due to an autoimmune process without obvious organic symptoms or paraclinical findings derived from the criteria for autoimmune encephalitis according to Cellucci et al. (2020). In this article, we briefly describe the criteria and a developed diagnostic pathway for autoimmune-based psychiatric symptoms in children (Fig. 2).

## Methods

We looked for articles listed in PubMed and published between 1988 and 2020 (23 September 2020) for the terms “psychiatry antibody children/pediatric/paediatric” or “psychiatry autoantibody children/pediatric/paediatric”. After this first screen, we searched Pubmed for the items “OCD children/pediatric/paediatric antibody” or “ADHD (attention deficit hyperactivity disorder) children/pediatric/paediatric antibody” or “autism children/pediatric/paediatric antibody” “Gilles de la Tourette syndrome (TS) children/pediatric/paediatric antibody “ or “psychosis children/pediatric/paediatric antibody” or “maternal transfer autoantibody children/pediatric/paediatric” or “catatonia psychiatry antibody children/pediatric/paediatric”. We reported relevant studies showing an association between serum or cerebrospinal fluid (CSF) autoantibodies and reported psychiatric disorders [psychosis, catatonia, TS, ADHD, OCD, autism spectrum disorder (ASD)] in our narrative review below.

## Results

### Maternal–fetal transfer of autoantibodies

We present animal and human research data from the literature concerning the maternal–fetal transfer of autoantibodies, which can severely affect the neurodevelopment of the fetus or newborn child, with consequences for later behavioral abnormalities potentially culminating in disorders such as ADHD or ASD. In general, maternal immunoglobulin G begins to cross the placenta at the beginning of the second trimester (Palmeira et al. 2012). The blood–brain barrier is not fully developed then. The fetal brain is thus theoretically susceptible to antibodies—even though the mother might be unaffected due to her functioning blood–brain barrier. So far, three groups of autoantibodies against membrane surface antigens have been identified in conjunction with maternal–fetal transfer: (1) *N*-methyl-d-aspartate receptor (NMDAR) antibodies (Wang et al. 2012; Jurek et al. 2019; Lee et al. 2009a), (2) Contactin*-*associated protein*-*like 2 (CASPR2) antibodies (Coutinho et al. 2017a, b; Brimberg et al. 2016; Bagnall-Moreau et al. 2020) and (3) antibodies against the 37 and 73 kDa proteins (Braunschweig et al. 2013). Furthermore, although our review focuses on membrane surface autoantibodies, we call brief attention to evidence of the maternal–fetal transfer of anti-Sjögren's-syndrome-related antigen A (SSA) (Ro) and anti-SSB (La) and anti-ribonucleoprotein 1 (RNP) autoantibodies in mothers with lupus erythematosus (Feki et al. 2015, Zurgil et al. 1993) to better decipher the spectrum of potential maternal–fetal transfer of autoantibodies.

#### Maternal–fetal transfer of N-methyl-D-aspartate receptors autoantibodies

Several studies Wang et al. (2012), Jurek et al. (2019), Lee et al. (2009a; b) addressed the effects of different NMDAR antibodies’ exposure of mice in utero. Wang et al. (2012) observed that specific NR2A-expressing neuronal apoptosis can be induced within the murine female brainstem after the application of double-stranded desoxyribonucleotide acid (DNA) antibodies that cross-react with the NMDAR’s NR2A and NR2B subunits. They demonstrated how these antibodies may enter brain parenchyma and result in neuronal brain damage. In addition to studying such neuropathological changes caused by infiltrating autoantibodies, Jurek et al. (2019) investigated the long-term behavioral consequences by assessing the behavioral reaction of mice after being given NMDAR antibodies during pregnancy. The transfer of human recombinant immunoglobulin 1 (IgG1) NR1 (GluN1) subunit of NMDAR antibodies resulted in long-term dysfunctional rodent behavior revealing rising mortality, sensory and motor impairment, and hyperlocomotion. In particular, hyperlocomotion in different rodent models could represent a cardinal feature of psychiatric disorders such as ADHD (Zimmermann et al. 2015) or mania (Souza et al. 2014). The rodent model is a surrogate model for the effects of NMDAR antibodies in humans suggesting structural and functional consequences following the maternal–fetal transfer of NMDAR autoantibodies and leading to behavioral abnormalities. Concerning humans, recent data [2010–2019 from Dalmau (2020)] indicate that neonatal mortality is low and children of mothers with NMDAR encephalitis do not suffer from further symptoms over time. Thus, the relevance of NMDAR antibodies in children is unclear and requires further investigation. Animal studies suggest an association between dysfunctional neurodevelopment and NMDAR antibodies, although the mechanism is still unclear. However, these results might implicate that a mother’s NMDAR encephalitis during pregnancy could lead to severe behavioral disability in her offspring, and enable an “autoimmune hit” during neuronal development.

#### Maternal–fetal transfer of contactin-associated protein-like 2 autoantibodies

Our literature research revealed studies Coutinho et al. (2017a, b), Brimberg et al. (2016), Bagnall-Moreau et al. (2020) demonstrating effects on brain structure and function by the maternal–fetal transfer of CASPR2 autoantibodies. Coutinho et al. (2017a, b) inspected the amount and distribution of glutamatergic synapses within the somatosensory and prefrontal cortex. They observed an alteration in the distribution of glutamatergic synapses within the somatosensory cortex. Furthermore, the numbers of glutamatergic synapses were decreased in the prefrontal and somatosensory cortex. Such aberrations in the composition and distribution of glutamatergic synapses are compatible with a hypothetical etiology of psychiatric disorders such as autism and schizophrenia (Coley and Gao 2018). To ignite brain inflammation, the activation of phagocytes in the central nervous system (termed microglia) seems to play a decisive role (Sousa et al. 2018). Coutinho noted more activated microglia in mice receiving CASPR2 antibodies compared to controls. Their findings indicate an immune activation responsible for persistent brain inflammation in mice. The upregulation of activated microglia and reduction (e.g. elimination) in glutamatergic synapses is a phenomenon called synaptic pruning. Inflated synaptic pruning has been associated with neurodevelopmental disorders such autism and schizophrenia (Neniskyte and Gross 2017). There is also evidence that knockdown of the CASPR2 (CNTNAP2) gene might affect dendritic spine density (as a crucial part of the neuron relevant for sufficient neurotransmission). Levels of glutamatergic receptors such as GluA1 subunits of AMPA receptors in murine dendritic spines might be altered (Varea et al. 2015), so that balanced excitatory glutamatergic neurotransmission might be disturbed. Furthermore, the altered dendritic morphology of excitatory neurons in the hippocampus is further corroborated by the maternal–fetal transfer from man to mice study, according to data from Brimberg et al. (2016). Their findings could imply substantial alterations within neuronal transmission. Alterations in neuronal excitatory transmission could lead to the generation of psychiatric symptoms such as psychosis via glutamatergic hypofunction as the “glutamate hypothesis” proposes (Kim et al. 1980). Furthermore, aberrant dendritic morphology is evident in neurodevelopmental disorders such as autism (Ma et al. 2019). In turn, CASPR2 autoantibodies could lead to structurally altered dendrites, thereby linking neurodevelopment disorders to initial autoimmunity with CASPR2 autoantibodies. In addition to structural abnormalities, deficiencies in cortical development, social capacities, learning, and repetitive behavior are observed in mice exposed in utero to monoclonal CASPR2 antibodies (Bagnall-Moreau et al. 2020). Furthermore, male mice in particular born to dams harboring polyclonal anti-CASPR2 antibodies exhibit the aforementioned abnormalities in cortical development and dendritic complexity of excitatory neurons, as well as behavioral deficits (Bagnall-Moreau et al. 2020). ASD demonstrates pronounced male predominance also; intriguingly, the CNTNAP2 mutation-related ASD phenotypes are also more frequent in males, suggesting a sex-associated susceptibility to anomalies in CASPR2 functioning (Alarcón et al. 2008; Bien et al. 2017). However, the role of CASPR2 autoantibodies in autism is controversial, as one study showed elevated CASPR2 autoantibodies in pregnant women who had children who were mental retarded, but not autistic (Coutinho et al. 2017a, b). In conclusion, the transfer of CASPR2 autoantibodies in rodents could result in major structural and functional deficits in neurodevelopment, suggesting the pathogenic potential of CASPR2 autoantibodies for human neuronal development.

#### Maternal–fetal transfer of fetal brain protein antibodies

In our literature search, we identified one study Bauman et al. (2013) that assessed the effect on monkeys of the maternal–fetal transfer of fetal brain protein antibodies with a molecular weight of 37 or 73 kDa. They investigated the behavior of macaques given IgG from mothers whose children were autistic (IgG-ASD) in their second or third trimester of pregnancy for a long 2-year period. The eight macaques with IgG-ASD exhibited inappropriate social interaction, namely more frequent contacts with familiar and unfamiliar peers than the controls. These findings could imply a more aggressive, impulsive and social not effective behavior of these macaques with IgG-ASD. Moreover, increased white matter volumes are observed in macaques with IgG-ASD, similar to the increased brain volume in young males with ASD (Nordahl et al. 2011). These results concur with abnormal behavior and also indicate structural abnormalities suggesting the induction of autistic behavior in macaques. These monkeys are very suitable for animal-model investigations, as their brain organization and social interaction reveal considerable similarities to us humans. In addition, another study confirms autistic behavior in mice with specific fetal brain autoantibodies (Jones et al. 2020) indicating antibody-mediated autoimmunity as a probable mechanism that might be species independent.

### Psychiatric syndromes and disorders in children associated with neuronal autoantibodies

The possible effects of a transfer of maternal autoantibodies to newborns could have consequences for the functional, intellectual, and social abilities of children as mentioned in animal and human studies above. Below we report on psychiatric syndromes and disorders associated with neuronal autoantibodies (Table 2).

#### Psychosis

Psychosis in children is characterized by obvious disturbances in their behavior, mental activity, and perception (for review see McClellan 2018). In 54% of 43 patients with first-episode psychosis, dopamine 2-receptor antibodies and NMDAR-receptor antibodies were detected in the children’s serum (Pathmanandavel et al. 2015) (Table 1). The differential diagnosis of psychosis is very important, as psychosis can be also a symptom of a basal ganglia encephalitis with dopamine receptor 1 (DR1) and DR2 antibodies in Syndenham chorea and PANDAS (Chain et al. 2020; Pollak et al. 2020). An animal study proved that autoantibodies from patients with Sydenham chorea target the DR2 on neurons (Cox et al. 2013) suggesting a pathogenic link between the evolution of psychotic symptoms and dopamine receptor autoimmunity. In other case series, patients presenting a first-episode psychosis revealed serum thyroid antibodies in conjunction with auditory and visual hallucinations as the predominant clinical features. CSF NMDAR antibodies are known to be associated with aggression, and there is evidence that serum LGI1 antibodies are associated with aggression and sleep disturbance (AlHakeem and Tabrki 2017). The presence of CSF NMDAR antibodies in patients suffering a first-episode psychosis suggests strong evidence of autoimmunity. Pediatric NMDAR encephalitis represents a large amount of all NMDAR encephalitis cases, and is usually associated with seizures and abnormal movements, while adults present more often with psychiatric disorders. Nevertheless, pediatric psychosis accompanying anti-NMDAR encephalitis has also been reported (Brenton et al. 2016): subtypes of psychosis might thus have an autoimmune origin in children. However, the latest evidence supports an autoimmune origin of psychosis in children associated with neural cell-surface autoantibodies only when additional clinical features or paraclinical findings of autoimmune encephalitis (Cellucci et al. 2020) occur in conjunction. The psychopathology of psychosis will be described in more depth in future studies to help us more accurately determine which patients require a sophisticated diagnostic approach to detect autoantibodies.

**Table 1:** Psychiatric disorders and psychiatric syndromes associated with autoantibodies in children

Disorders/symptoms	*n*	ABS associated	Material	Test	References
Psychiatric disorders/syndromes					
Psychosis	9/43	DR2, NMDAR, LGI1	Serum	fCyt, live CBA	Pathmanandavel et al. (2015)
ADHD	4/15	GAD65	Serum	ELISA, immunohistochemistry mice brain tissue	Rout et al. (2012)
	15	DAT	Serum	ELISA	Giana et al. (2015)
ASD	2/37, 5/37, 11/37	37, 39, 73 kDa	Serum	Western blot, rhesus macaque brain tissue	Rossi et al. (2013)
	95/355	45, 62 kDa	Serum	Western blot, rhesus macaque brain tissue	Piras et al. (2014)
	20	Frontal cortex	Serum	Radial immunodiffusion assay	Todd et al. (1988)
	3/20	GAD65	Serum	ELISA, immunohistochemistry mice brain tissue	Rout et al. (2012)
OCD	7/21	55, 86 kDa	Serum	Immunoblot, immunohistochemistry	Morer et al. (2008)
	21/50	ABGA	Serum	Western blot, ELISA	Dale et al. (2005)
	261/311	DR1, LG	Serum	ELISA, human neuronal cell line	Cox et al. (2015)
Mental retardation	1/11	CASPR2	Serum	Live CBA	Coutinho et al. (2017a, b)

*ABGA* anti basal ganglia antibodies, *ADHD* attention deficit hyperactive disorder, *AMPAR* α-amino-3-hydroxy-5-methyl-4-isoxazolepropionic acid, *CASPR2* contactin-associated protein 2, *CBA* cell-based assay, *DR2* dopamine receptor 2, *ELISA* enzyme linked immunosorbent assay, *fCyt* flow cytometry, *GAD65* glutamic acid decarboxylase 65, *n* number, *NMDAR* *N*-methyl-d-aspartate receptor, *PC* Purkinje cell, *VGKC* voltage gated potassium channel

#### Attention-deficit hyperactivity disorder

Attention-deficit hyperactivity disorder (ADHD) is a disorder in children whose clinical features are inattention, disorganization, or hyperactivity-impulsivity; it constitutes a frequent neurodevelopmental disorder that can persist into adulthood (for review see Cabral et al. 2020). GAD65 antibodies were detected in 27% of 15 children with ADHD, but in none of the controls. However, as the titer of antibodies did not correlate with mental retardation (Rout et al. 2012) and the GAD65 antibodies were detected in serum, the evidence for autoimmunity is low. Dopamine transporter (DAT) autoantibodies are a potential marker of the psychopharmacological treatment response, as basal DAT antibodies are elevated in untreated patients with ADHD (Giana et al. 2015). Considered together, there is little evidence from subgroups of ADHD patients with potentially autoimmune-mediated symptoms. More thorough investigation is necessary to further clarify the pathogenic relevance of these serum antibodies.

#### Autism spectrum disorders

Autism spectrum disorders (ASD) in children are defined by repetitive behavior or activities culminating in social-interaction deficits (for review see, Mughal et al. 2020). There is low to moderate probability for autoimmunity in ASD, as made evident through studies showing proven serum autoantibodies to brain proteins [often not specified, i.e., antibodies against proteins 37, 39, 45, 62, 73 kDa (Rossi et al. 2013; Piras et al. 2014)]. 45 kDa protein antibodies are known to correlate with autism severity, as indicated by cognitive impairment and lower scores on behavioral scales in the Piras et al. study (2014), suggesting that specific brain autoantibodies play a pathophysiological role in inducing or exacerbating autistic symptoms. That assumption is further corroborated by their findings (Piras et al. 2014) that maternal 37, 39 and 73 kDa autoantibodies correlate with the verbal and non-verbal capacities of ASD children, and that the 62 kDa autoantibody is associated with more stereotypical behavior. Furthermore, we noted serum anti-brain antibodies against the human frontal cortex in a mentally retarded group of ASD patients compared to depressed controls (Todd et al. 1988) implicating a possible role of frontal cortex immunity in ASD. However, the evidence for such autoimmunity located within the frontal cortex is low, and further investigations entailing CSF analyses are needed. Interestingly, the evidence of elevated specific serum antibodies such as antibodies against lactate dehydrogenase (LDH), stress-induced phosphoprotein 1 (STIP1), collapsin response mediator protein 1 (CRMP1), or cypin from mothers with ASD children displaying stereotypical behaviors (Braunschweig et al. 2013) adds credence to the relationship between the maternal–fetal transfer of autoantibodies and induction of autistic behavior. The role these autoantibodies play in worsening neurodevelopment is further supported by the role they play in cell migration (Braunschweig et al. 2013), apoptosis (Charrier et al. 2006), gray matter integrity (Yum et al. 2017) and dendritic organization (Patel et al. 2018; Ariza et al. 2017). A major challenge is to characterize these aforementioned and other autoantibodies, thereby confirming their pathogenic relevance through CSF studies, and to delineate their role better within the induction of autistic behavior.

#### Obsessive–compulsive disorder

Obsessive–compulsive disorder (OCD) is defined by obsessions in combination with compulsions that mildly to severely impair the patient´s quality of life (for review see, Nazeer et al. 2020). Increased serum antibodies against basal ganglia (ABGA), Dopamin 1 receptor (DR1) and lysoganglioside have been detected in patients with OCD compared to controls (Dale et al. 2005; Cox et al. 2015). However the pathogenicity of ABGA antibodies is highly doubtful (Dale and Brilot 2012). Furthermore, OCD associated with membrane surface autoantibodies must be distinguished from PANS. It is worth carrying out further studies involving CSF analysis to disentangle the significance of these autoantibodies regarding symptoms and disease generation.

#### Tics and Gilles de la Tourette syndrome

Tics and Gilles de la Tourette syndrome (TS) are hyperkinetic movement disorders in childhood. Tics appear suddenly and are non-rhythmic, involving often repetitive motor movements or phonic tics (vocalizations), whereas TS is characterized by both motor and phonic tics persisting for more than a year (for review see Mittal 2020). Autoantibodies against two not-further-specified proteins (55, 86 kDa) were identified in 21 patients with TS (Morer et al. 2008). There are no investigations that analyzed CSF autoantibodies in children with TS.

#### Catatonia

Catatonia is characterized by immobility and stupor, posturing, mutism and waxy flexibility, echolalia, and an excitatory phase characterized by bizarre, non-goal-directed hyperactivity. This syndrome has been historically considered as pathognomonic for schizophrenia, but it is also present in other psychiatric (e.g., depression) and neurological disorders. Immunological processes are increasingly implicated as causes for catatonia in the field (Rogers et al. 2019). Pediatric catatonia is defined as “organic” in approximately 20% of patients, and is also associated with PANDAS and pediatric autoimmune encephalitis (Lahutte et al. 2008). Antibody-mediated catatonia has not just been diagnosed—it has also been treated successfully in children with systemic lupus erythematosus, anti-NMDAR encephalitis (Consoli et al. 2012) and GABA_A_ receptor encephalitis (Nikolaus et al. 2018). Importantly, catatonia in these cases (Consoli et al. 2012) was accompanied by polysymptomatic neurological abnormalities like seizures, movement disorders and by multisystemic features of systemic lupus erythematosus. Screening for autoantibodies against neuronal surface antigens and CSF analysis should thus be considered in children suffering from catatonia.

#### Igniting an autoimmune attack in children

We have reported on observing the fetal transfer of maternal neuronal autoantibodies and consequences for dysfunctional behavior and brain damage in animal models. Furthermore, we have reported psychiatric syndromes or disorders associated with serum neuronal autoantibodies in children. NMDAR, CASPR2 as well as 37 Da and 73 k Da fetal brain autoantibodies are believed to be pathogenic as these antibodies constitute cell-surface antibodies. Antibody studies have pointed out the pathogenicity of NMDAR (Malviya et al. 2017) and CASPR2 (Saint-Martin et al. 2019) in humans. A probable autoimmune disease was induced in animal models (Wang et al. 2012; Jurek et al. 2019; Lee et al. 2009a, b; Coutinho et al. 2017a, b; Brimberg et al. 2016; Bagnall-Moreau et al. 2020) as tissue investigations and behavioral analysis, suggesting a pathogenic role of these autoantibodies. However, as psychiatric symptoms are difficult to assess in animals, specific symptoms such as hyperlocomotion can be interpretated as surrogate behavior suggesting, for example, mania or ADHD like symptoms. Thus, keeping the difficulty of assessing psychiatric symptoms in animals in mind, we can reproduce a disease caused by the transfer of autoantibodies triggering brain damage and inducing abnormal behavior. Moreover, we have clinical hints that children, like those with NMDAR antibodies and psychosis (Pathmanandavel et al. 2015) and children with CASPR2 antibodies and mental retardation (Coutino et al. 2017a, b) and 37 and 73 kDa antibodies against fetal brain antibodies in combination with ADHD symptoms (Rossi et al. 2013) might bear clues for an autoimmune disease associated with behavioral abnormalities. We thus believe that NMDAR, CASPR2 and 37 and 73 kDa against fetal brain protein antibodies are pathogenic. Taken together, Whitebsky’s revised criteria for an autoimmune disease are fulfilled, and reflect the pathogenicity of these autoantibodies in children (Rosa and Bone 1993). However, there have been no studies assessing the long-term effects of autoimmune encephalitis or antibody-associated psychiatric syndrome over years or decades to evaluate whether such an initial hit could be the trigger for the initial manifestation of psychiatric disorders. Furthermore, a recent study by Hammer et al. (2014) found that the antibody presence in schizophrenia patients was associated with a history of birth complications or brain injury. It is therefore conceivable that brain injury could lead to NMDAR antibody seropositivity. However, the pathogenic role of NMDAR antibodies in the development of schizophrenia is not supported by their findings. Moreover, they detected no phenotypic differences in patients with schizophrenia with or without NMDAR antibodies in their study. NMDAR antibodies are believed to be pathogenic in NMDAR encephalitis (Malviya et al. 2017) and in NMDAR antibody-associated psychosis via a reorganization of synaptic NMDARs (Jézéquel et al. 2017). Further longitudinal studies in transition psychiatry in children with early neuroglial autoantibodies in their lifetime will have to be conducted to find out how relevant these neuroglial antibodies in early childhood may be in later adult life in generating psychiatric symptoms.

There are autoimmune encephalopathies characterized by their chronic relapse-remitting character, such as GAD65 autoimmune encephalitis (Hansen et al. 2018), which probably disturbs brain function and structure repeatedly and may lead to permanent deficits resulting in the manifestation of psychiatric disease. Furthermore, a recent report suggested that a patient who recovered from LGI1- encephalitis developed a new onset psychotic disorder after surviving a one-year course of LGI1-antibody-positive encephalitis (Pollak and Moran 2017). This is a paradigmatic example of how a subsiding or chronic, recurrent autoimmune encephalitis with circulating serum or cerebrospinal fluid autoantibodies may influence psychiatric disorders. However, the underlying mechanism of circulating serum autoantibodies that may permeate the blood–brain barrier temporarily due to a transient dysfunction of the blood–brain barrier in early childhood must be differentiated from that of the maternal–fetal transfer of brain autoantibodies. However, if the blood–brain barrier is impervious, the underlying mechanism remains obscure in children with circulating serum autoantibodies. We postulate that both mechanisms [(a) fetal transfer and (b) circulating autoantibodies] contribute to our “autoimmune-priming-attack hypothesis”. An early autoimmune process during autoimmune encephalitis, autoimmune psychosis (Pollak et al. 2020), autoimmune dementia (Flanagan et al. 2010) or an antibody-associated psychiatric syndrome (Hansen et al. 2020) could occur at a critical stage in human neurodevelopment, thereby affecting either the structure or function of CNS development. Thus, although the autoimmune process appears to recover (verified by the lack of autoantibodies), residual structural and functional deficits may be sowing the breeding ground for the development of specific subgroups of psychiatric disease. Furthermore, impaired brain development due to circulating antibodies could be a risk factor for a later psychiatric disease onset in case of a “second hit” (e.g., drug consumption, life events, etc.). Our “autoimmune-priming-attack hypothesis” could be especially relevant for behavioral disorders such as ASD or ADHD, but also psychotic disorders. In the next section, we report on several autoantibodies-associated psychiatric disorders in childhood.

### Autoantibody-based psychiatric syndrome in children

We recommend classifying the appearance of psychiatric symptoms lasting under 3 months as subacute psychiatric syndrome (aPS), and those lasting more than 3 months as a subchronic psychiatric syndrome (cPS) providing additional criteria are fulfilled (Fig. 2) in analogy to previously published criteria according to Hansen et al. for adults (2020). Furthermore, these criteria are based on recently published criteria for autoimmune encephalitis in children (Cellucci et al. 2020). A possible autoimmune a/cPS (a/cAPS) should be presumed if a subacute psychiatric syndrome is present in addition with the existence ≥ 2 indicators of autoimmunity listed in Table 2. To assume a probable a/c APS ≥ 1, features in paraclinical investigations must be fulfilled. These criteria indicate an autoimmune origin and comprise additional items based on an inflammation (pleocytosis with ≥ 5 /µl or intrathecal IgG synthesis) in the cerebrospinal fluid (CSF), focal or generalized epileptic potentials or focal slowing in the electroencephalography (EEG) and/or features often found in the temporal lobe in magnetic resonance imaging (MRI) investigations indicating encephalitis (Fig. 2) or via an inflammation detected in a brain biopsy after excluding other diseases. Autoantibody positivity in serum or CSF leads to the occurrence of a definitive a/cAPS if ≥ 1 paraclinical findings are present. However, if MOG, NMDAR or GAD65 antibodies are found in the CSF, no further paraclinical findings are important to diagnose a definitive a/cAPS. The presence of serum neural cell-surface autoantibody positivity is not necessary to assume a probable a/cAPS. The no-proof-of-neuroglial-autoantibodies condition in conjunction with other clinical and paraclinical features suggesting probable a/cAPS is termed seronegative a/cAPS. Further markers suggest brain damage, but are not marker-established (such as elevated neuroglial proteins) for diagnosing an APS. Furthermore, CSF neopterin in children seems to be a promising marker for neuroinflammation (Molero-Luis et al. 2020). We recommend searching for a specific autoantibody panel in serum and CSF according to suggested antibodies for autoimmune pediatric encephalitis (Cellucci et al. 2020) and considering results from our literature research. The autoantibody panel consists of autoantibodies against NMDAR, MOG, GAD65, GABAAR for initial screening, and if those are negative but there are further hints suggesting an autoimmune condition, the antibodies that should be determined are: ABGA, CASPR2, LGI1, DAT, DR2, DR1, mGluR5, Glycin and GABABR. The testing in serum and CSF is necessary, as the sensitivity for detecting antibodies differs between serum and CSF depending on the autoantibody being tested. In addition, we advise using cell-based assays for serum and CSF antibody testing when searching for antibodies against membrane surface antigens and enzyme-linked immunosorbent assays (ELISA) combined with absorption-spectrometry when seeking GAD65 antibodies. The antibody’s relevance is assessed in the second step via tissue-based systems; immunofluorescence and immunohistochemistry should confirm the detected autoantibodies from the first step. Differential diagnoses and ruling out other diagnoses are important steps in diagnosing APS. Careful exclusion of differential diagnoses must be undertaken, considering brain trauma, systemic inflammatory and primary CNS inflammatory disorders such as infectious encephalitis, infectious meningoencephalitis or infection-associated encephalopathy, diseases with postulated immune mechanisms, malignancies, intoxications, nutritional, neoplastic, metabolic, psychiatric or endocrine disorders, electrolyte disturbances, epilepsy as well as nonconvulsive status epilepticus.

**Table 2:** Important clinical features as indicators for autoimmune involvement in children

Acute regression in childhood development
Altered level of consciousness
Altered mental status
Catatonia
Cognitive dysfunction
Dynamic psychopathology
Focal neurological deficits
Lack of treatment response (antidepressants, antipsychotics)
Movement disorder
Psychiatric symptoms
Seizures not explained by a known epilepsy

Complemented and modified from Cellucci et al. (2020)

## Discussion

### Synopsis: autoantibody-associated psychiatric syndromes in children

We identified six major groups of psychiatric disorders known to be associated (and appearing in subgroups) with diverse serum neuronal autoantibodies. Further research entailing the investigation of specific antibodies, clinical features (Table 2) and inflammation markers in CSF is required to be able to claim a probable autoimmune etiology in subgroups of these patients.

### Diagnostic aspects

We recommend promoting CSF testing in the aforementioned patient groups, as it is currently the only way to prove IgG autoantibodies in the CSF of definitive autoimmune origin (Fig. 2). As an initial approximation serum analysis could be helpful, if lumbar puncture is not accessible or tolerable. Nevertheless, we advise its use for severely affected patients presenting these assumed diagnoses: psychosis, catatonia, ASD, OCD, ADHD, TS or other tics. Furthermore, the symptoms below should lead clinicians to carry out more thorough diagnostics as exemplified in Fig. 2: psychosis, inattention, hyperactivity, impulsivity, obsessions, compulsions, motor and phonic tics as well as catatonia.

### Ethical considerations

Although no evidence of CSF autoantibodies-associated ASD, OCD, ADHD, TS, catatonia or other tics has been identified to date, we recommend performing CSF analysis to detect IgG antibodies establishing a more probable autoimmunity. However, important issues must be carefully considered beforehand, namely a thorough consent procedure and consultation clarifying its main benefits and risks. The child’s parent or parents or legal guardian would have to agree and provide written consent. Furthermore, immunotherapy in children with autoimmune encephalitis (for review, see Garg et al. 2020; Zuliani et al. 2019) and autoimmune-based psychiatric syndromes is an optional therapy whose evidence level is low to moderate (evidence level IV through case control and cohort studies). However, it is not within the scope of our article to make recommendations for specific treatment, as no meta-analyses or randomized placebo control studies have been conducted in autoantibody-based psychiatric syndromes in children. Before administering immunotherapeutic treatment, the child’s custodian again would have to give their written permission and be informed that this is an experimental treatment given on an individual therapeutic trial basis.

### Limitations

The clinical significance of the reported neural serum antibodies associated with psychiatric disorders is unknown and questionable apart from the NMDAR antibodies believed to be pathogenic. GAD65 antibodies are described in children presenting well-known clinical disorders such as stiff-person syndrome, temporal lobe epilepsy or cerebellar ataxia, but much less is known about their occurrence in pediatric psychiatric syndromes. Their pathogenic relevance is dependent on high serum and CSF titers (Cellucci et al. 2020). Further large-scale studies are required to validate the clinical significance of these antibody findings. Although the evidence level is low and no descriptions about which antibody tests were made, we cited a single case from Al Hakeem et al. (Al Hakeem and Tabarki 2016) mentioning IgG NMDAR antibodies in CSF in association with aggression. Further studies in large patient cohorts are necessary to validate and confirm these preliminary findings.

## Conclusions

The maternal–fetal transfer of autoantibodies during gestation may be how a mother’s autoimmune encephalitis can trigger an autoimmune hit in her child (“autoimmune-priming-attack hypothesis”). Autoantibody-associated psychiatric disorders in childhood are subentities of psychosis, ADHD, ASD, OCD, catatonia or TS. We have created a clinical pathway based on our literature review indicating when to use specific diagnostic methods for identifying patients presenting underlying autoimmunity.
